# Risk factors for house-entry by malaria vectors in a rural town and satellite villages in The Gambia

**DOI:** 10.1186/1475-2875-7-2

**Published:** 2008-01-07

**Authors:** Matthew J Kirby, Clare Green, Paul M Milligan, Charalambos Sismanidis, Momadou Jasseh, David J Conway, Steven W Lindsay

**Affiliations:** 1 School of Biological and Biomedical Sciences, Durham University, Science Laboratories, South Road, Durham DH1 3LE, UK; 2 London School of Hygiene and Tropical Medicine, Keppel Street, London WC1E 7HT, UK; 3 Medical Research Council Laboratories, Fajara, PO Box 273, The Gambia

## Abstract

**Background:**

In the pre-intervention year of a randomized controlled trial investigating the protective effects of house screening against malaria-transmitting vectors, a multi-factorial risk factor analysis study was used to identify factors that influence mosquito house entry.

**Methods:**

Mosquitoes were sampled using CDC light traps in 976 houses, each on one night, in Farafenni town and surrounding villages during the malaria-transmission season in The Gambia. Catches from individual houses were both (a) left unadjusted and (b) adjusted relative to the number of mosquitoes caught in four sentinel houses that were operated nightly throughout the period, to allow for night-to-night variation. Houses were characterized by location, architecture, human occupancy and their mosquito control activities, and the number and type of domestic animals within the compound.

**Results:**

106,536 mosquitoes were caught, of which 55% were *Anopheles gambiae sensu lato*, the major malaria vectors in the region. There were seven fold higher numbers of *An. gambiae s.l*. in the villages (geometric mean per trap night = 43.7, 95% confidence intervals, CIs = 39.5–48.4) than in Farafenni town (6.3, 5.7–7.2) and significant variation between residential blocks (p < 0.001). A negative binomial multivariate model performed equally well using unadjusted or adjusted trap data. Using the unadjusted data the presence of nuisance mosquitoes was reduced if the house was located in the town (odds ratio, OR = 0.11, 95% CIs = 0.09–0.13), the eaves were closed (OR = 0.71, 0.60–0.85), a horse was tethered near the house (OR = 0.77, 0.73–0.82), and churai, a local incense, was burned in the room at night (OR = 0.56, 0.47–0.66). Mosquito numbers increased per additional person in the house (OR = 1.04, 1.02–1.06) or trapping room (OR = 1.19, 1.13–1.25) and when the walls were made of mud blocks compared with concrete (OR = 1.44, 1.10–1.87).

**Conclusion:**

This study demonstrates that the risk of malaria transmission is greatest in rural areas, where large numbers of people sleep in houses made of mud blocks, where the eaves are open, horses are not tethered nearby and where churai is not burnt at night. These factors need to be considered in the design and analysis of intervention studies designed to reduce malaria transmission in The Gambia and other parts of sub-Saharan Africa.

## Background

To achieve a reduction in malaria morbidity, the design and implementation of control strategies must be based on a sound understanding of the risk factors that contribute to transmission at a variety of spatial scales [1]. These include topography [2,3], proximity to mosquito breeding sites [4], house design [5], density of human populations [6], use of vector control methods [7-9], presence of domesticated animals [10], as well as the variation in attractiveness between individual human subjects [11,12] and their socio-economic status [9]. Of all these variables, malaria risk is perhaps most strongly influenced by household factors, which can account for about 28% of the total variability in incidence [13]. Identifying and tackling the household effects must, therefore, be an efficient route to reducing the burden of disease in malaria-endemic areas.

A multi-factorial risk factor analysis study was designed to highlight important spatial, compound-, house- and mosquito control-related parameters that affect house entry of malaria vectors in The Gambia. This study was carried out largely to identify important confounders that influence house-entry by mosquitoes; important information for the design and analysis of a randomized controlled trial to assess whether house screening can substantially reduce exposure to malaria vectors in the study area.

The main malaria vectors in The Gambia are members of the *Anopheles gambiae s.l*. species complex, namely *An. gambiae s.s*., *Anopheles arabiensis *and *Anopheles melas *[14,15]. These vectors are usually nocturnal and endophagic in their feeding behaviour [15,16] and, therefore, seek entry to dwellings occupied by humans at night. Thus much of the malaria transmission in this setting takes place at home, with around 80% occurring indoors [17].

A range of different factors known, or thought likely, to affect the number of *An. gambiae s.l*. caught indoors in The Gambia, were examined. These included the month of collection during the rainy season, the period of peak transmission [15,17], rural or urban location [18], the presence of cattle or horses in the compound [19], the type of house construction [5], the number of people in the room where the trap was hung, and methods of personal protection used by householders[17]. This is the first study to examine all these factors simultaneously in an area with both rural and peri-urban housing.

## Methods

### Study area

The study area was situated approximately 170 km from the mouth of the River Gambia and covered 70 km^2 ^of the North Bank Division in The Gambia, an area of open Sudan savanna vegetation. The climate consists of a single rainy season from June to October followed by a long dry season. Specifically, the study area comprised 976 houses; 539 houses in 11 residential blocks in Farafenni town (UTM coordinates: 1500200N, 435500E) and 437 in 16 villages located to the south and east, within 5 km of the town. Blocks and villages were selected that were accessible within 20–30 minutes of MRC station, where good demographic data was available, and that had participated in previous MRC studies with good levels of compliance. Farafenni itself is a market town with a population of over 20,000 inhabitants, situated less than 5 km from the River Gambia and 2 km south of the border with Senegal. Houses close to the town centre typically have three or more rooms with concrete or mud brick plastered walls and metal roofs. On the outskirts of the town the houses more closely resemble those in the rural villages, characterized by a single-room with unplastered mud brick walls, a thatched roof and open eaves. Houses in both the town and villages are usually arranged in familial compounds demarcated by a fence or wall, though there are some in which the houses are rented by unrelated family groups. Compounds in Farafenni contain typically one to four houses and in the villages 4–6 houses, but sometimes as many as 20. All houses studied were part of a demographic surveillance system (FDSS) that incorporates 46 residential blocks in the town and 23 surrounding villages, also defined as individual blocks within the FDSS.

The study population comprised 5848 people dominated by three ethnic groups; Mandinka (38%), Wollof (31%) and Fula (23%). Mandinkas represent 50% of the Farafenni study population but only 20% of those are from the villages. There are roughly equal numbers of men and women both in Farafenni and in the villages.

### Mosquito collections

976 houses were each sampled on a single night between 17 August and 25 October 2005. Over 97% of all houses in each block were included in the study. A trapping room was selected where a single person slept and where there was no ceiling and the eaves were at least partially open. If that was not possible, we selected the room with the fewest number of people. Light traps were also hung in four sentinel houses, two in Farafenni, one in a village to the east, Kunjo, and one in a village to the south, Duta Bulu, on every night of collection in order to adjust for night-to-night variation in mosquito catches. These houses were occupied by a single male adult that slept under an untreated bednet for the duration of the rainy season.

A CDC miniature light trap (model 512) was positioned 1 m above the ground within 1–2 m of the foot end of a bed protected with a new untreated bednet provided on that night only. If the trapping room contained multiple beds, then the other room occupants were encouraged to use their existing bednets. If they had none, additional new bednets were provided for that night. Light traps operated from 19:00 (min, 19:29 max) to 07:00 (min, 07:53 max) the following morning. Mosquitoes were killed by freezing at -25°C for two hours and identified using morphological criteria. 1% of the *An. gambiae s.l*. complex caught in the traps was identified to species by PCR analysis [20].

### Putative risk factors

Spatial parameters recorded were house location (block and urban/rural). Compound parameters recorded were the number of cows and horses in the compound between 19.00 and 07.00. Variables of house structure recorded were roofing material, wall material, number of storeys, house external length and width, trapping room internal length, width and height, eaves open/closed, size of eave gap, presence or absence of ceilings, presence or absence of screening, presence or absence of electric lights. Variables of occupants recorded were the age, sex and ethnicity of those sleeping in the bed nearest the light trap, the number of children sleeping in the trapping room and all other rooms on the night of trapping, the number of adults sleeping in the trapping room and all other rooms on the night of trapping. Variables of mosquito control recorded were use of mosquito coils, local incense (churai), insecticide spray and insecticide-treated nets within the trapping room and/or all other rooms on the night of trapping.

### Environmental and weather records

In the trapping room of four houses and one sentinel house in Farafenni, each night from 7 September to 25 October, a single data logger (HOBO^® ^U12 Temp/RH/Light External Data Logger) was hung from the wall or put in a dry place not on the floor, near the light trap. The loggers were pre-set to turn on automatically at 19:00. Loggers were collected each morning at 07:00, switched off, and the data downloaded to Onset HOBOware™ version 2.0 software. In addition, daily minimum and maximum temperature and relative humidity data and rainfall were acquired from Yallal meteorological station, approximately 15 km west of Farafenni.

### Statistical analysis

**Figure 1:**
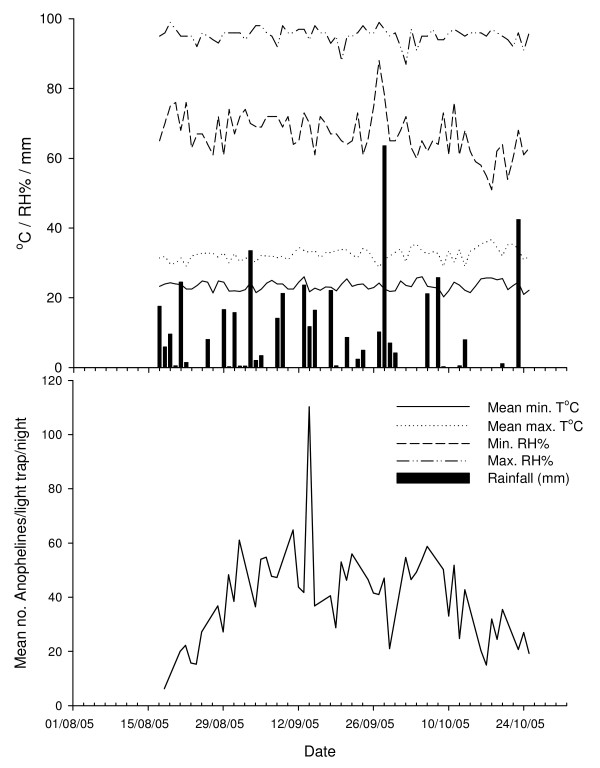
Mean number of *An. gambiae s.l*. caught from four sentinel houses and weather conditions during the study.

Mosquito catches from individual houses were analysed as unadjusted counts, and adjusted to cater for the night to night variation in mosquitoes in the sentinel houses (Fig. 1). Mosquito catches from study houses were adjusted to be proportional to the sentinel house mean (expressed as 1) using the following formula:

Adjusted catch in house x = actual catch in house x_t_/(catch in st1_t _+ st2_t _+ st3_t _+ st4_t_)/4)

Where x_t _represents the catch from a target house on one night and st1_t_, st2_t_, st3_t _and st4_t _represent the four collections from the sentinel houses on the same night.

For some variables there were too few values to determine whether they affected mosquito house entry or not (evidence of screening n = 23; electric lights, n = 7; multi-storey housing, n = 1) and were not analysed further. Friedman test was used to check consistency between sentinel house sites. Kruskal Wallis test was used to explore differences in mosquito prevalence between blocks and a Mann-Whitney U test was used to look for an urban-rural division.

The unconditional variance of the mosquito counts (SD = 153) in the 976 houses was much larger than the mean (mean = 60), therefore a negative binomial model was used that, unlike the Poisson model, accounts for the excess variation. In this model, the mosquito count variable is assumed to be generated by a Poisson-like process, except that the variation is greater than that of a true Poisson (overdispersion).

The association of each risk factor with mosquito catch size was individually assessed with univariate analyses. If the univariate regression model for a given covariate resulted in a P-value greater than 0.1 then this covariate was excluded from the multivariate investigations. Where plausible, the risk factors were categorized into four groups within which there was a high degree of correlation. Location was incorporated in the model, but not as part of a specific group. The four groupings were:

1. Number of children in the trapping room, people in the trapping room, children in the house, people in the house, house external length and width, trapping room internal length, width and height.

2. Wall type, roof type, eaves (presence/absence), size of eave gap

3. Use of mosquito coils, local incense (churai), insecticide spray and insecticide-treated nets

4. Number of cows and horses within the compound

The multivariate analysis was conducted in two steps. Firstly the association of the outcome with groups of risk factors described above was investigated. This eliminated some of the risk factors and represented a group of highly correlated variables by possibly just one variable. In step II all significantly associated variables with the outcome from step I as well as those that did not fit in a group were included in the model. Variables were retained in this model if their association with mosquito count was significant (p < 0.05). Urban/rural classification is a known important factor and, therefore, remained in the model throughout the multivariate analyses. The final multivariate model was built twice, once with and once without adjustment for the sentinel catch. The seasonal variation of mosquito counts was also investigated, by adding month as a factor in the model, but seasonality did not affect the model, probably because most of the mosquito catch came from one month only (September).

### Ethics

Ethical approval for this study was given by the Gambian Government and Medical Research Council Laboratories Joint Ethical Committee and the Ethics Advisory Committee of Durham University.

## Results

### Mosquito numbers

106,536 mosquitoes were caught in 976 light trap collections during the 2005 rainy season, of which 66,129 (62%) were Anophelines and 58,729 (55%) *An. gambiae sensu lato*. Other Anopheline species caught were *Anopheles ziemanni, Anopheles squamosus*, *Anopheles pharoensis *and *Anopheles rufipes*. The common culicines were *Culex thalassius*, *Culex quinquefasciatus*, *Aedes aegypti*, *Aedes vittatus *and *Mansonia africanus*. Only three traps failed during the period; those houses were re-sampled within the sampling timeframe. Of the *An. gambiae s.l*. specimens identified by PCR, 43% were *An. gambiae sensu stricto*, 29% *An. arabiensis *and 28% *An. melas *(Table 1). The proportions of *An. gambiae s.s*. and *An. arabiensis *increased over the study period whilst that of *An. mela*s declined (χ^2 ^for trend = 15.87, df = 2, p < 0.001, Table 1).

**Table 1:** Relative proportion of *Anopheles gambiae *species complex members in random monthly samples from light trap catches.

Month	*An. gambiae s.s*. (%)^a^	*An. arabiensis *(%)^a^	*An. melas *(%)^a^	Total	No result^b^	Total PCR specimens	% of total catch identified by PCR
Aug	25 (38)	16 (25)	24 (37)	65	6	71	0.9
Sept	75 (40)	48 (25)	67 (35)	190	21	211	0.5
Oct	93 (51)	58 (32)	30 (17)	181	18	199	2.1

Total	193 (43)	122 (29)	121 (28)	436	45	481	0.8

^a ^proportion of each species divided by the total number of *An. gambiae s.l*., expressed as a percentage; ^b ^no amplification after 3 PCR attempts.

### Seasonal variation

Mosquito numbers in the sentinel houses rose from the beginning of the study to a plateau followed by a decline in October, with large variations in mosquito numbers from night to night (Figure 1). Greatest numbers of mosquitoes were collected in September and appeared unrelated to daily temperature, relative humidity or rainfall (Figure 1).

### Data validation

The rank order of attractiveness of the four sentinel houses did not vary during the study (Friedman test χ^2 ^= 97.7, N = 48, df = 3, p < 0.001), suggesting that the relative attractiveness of any study house remained the same over the trapping period.

### Vector risk factors

Mosquito numbers were considerably greater in rural houses compared to those situated on the edge of Farafenni town (Table 2). Risk factors operating at the compound level were associated with being far from a pit latrine and having cows next to the house at night. The presence of horses was associated with lower risk. Houses with mud brick walls, thatched roof, large open eaves with small rooms and large numbers of occupants were likely to have larger numbers of mosquitoes than those without these features. The use of churai, mosquito sprays and coils reduced the number of mosquitoes in a bedroom, but the presence of an insecticide-treated bed net did not affect mosquito numbers.

**Table 2:** Association between mosquito counts and potential risk factors as measured in incidence rate ratios (IRR) from negative binomial regression models.

		**Univariate**		Multivariate with sentinel* (N = 950)		Multivariate without sentinel* (N = 949)	
		**IRR (95%CI)**	**P-value**	**IRR (95%CI)**	**P-value**	**IRR (95%CI)**	**P-value**

*Sentinel*		1.02(1.013,1.025)	<0.0001	1.013(1.009,1.018)	<0.0001		
*Month of mosquito collection*							
	August	1	<0.0001				
	September	2.24(1.74,2.88)					
	October	0.67(0.52,0.87)					
*Location*							
	Rural	1		1		1	
	Urban	0.11(0.09,0.13)	<0.0001	0.11(0.09,0.13)	<0.0001	0.11(0.09,0.13)	<0.0001
*Distance (m) to nearest pit latrine*		1.006(1.003,1.01)	<0.0001				
*Cows in compound*		1.17(1.04,1.33)	0.012				
*Horses in compound*		0.92(0.85,0.99)	0.038	0.78(0.74,0.83)	<0.0001	0.77(0.73,0.82)	<0.0001
*Wall type*							
	Cement	1				1	
	Mud Brick	5.36(3.92,7.31)	<0.0001			1.44(1.10,1.87)	0.007
*Roof type*							
	Metal	1					
	Thatch	1.15(0.94,1.41)	0.163				
*Eaves*							
	Open	1		1		1	
	Closed	0.38(0.32,0.46)	<0.0001	0.73(0.62,0.86)	<0.0001	0.71(0.60,0.85)	<0.0001
*Eave gap size (cm)*		1.06(1.04,1.08)	<0.0001				
*House volume (m*^3^*)*		0.997(0.996,0.998)	<0.0001	.9988(.9978,.9997)	0.013		
*People in house*		1.03(0.99,1.06)	0.059	1.06(1.03,1.09)	<0.0001	1.04(1.02,1.06)	0.001
*Children in house*		1.05(0.99,1.12)	0.073				
*Trapping room volume (m*^3^*)*		1.02(1.01,1.03)	<0.0001				
*People in trapping room*		1.25(1.18,1.33)	<0.0001	1.17(1.11,1.24)	<0.0001	1.19(1.13,1.25)	<0.0001
*Children in trapping room*		1.16(1.05,1.28)	0.003				
*Sleepers in trapping bed*		1.19(1.06,1.34)	0.004				
*Coil in room*							
	No	1					
	Yes	0.47(0.35,0.62)	<0.0001				
*Insecticide spray in room*							
	No	1	<0.0001				
	Yes	0.14(0.05,0.37)					
*Churai in room*							
	No	1		1		1	
	Yes	0.50(0.40,0.62)	<0.0001	0.56(0.48,0.67)	<0.0001	0.56(0.47,0.66)	<0.0001
*Insecticide treated nets in room*							
	No	1	0.134				
	Yes	0.82(0.63,1.06)					

*Sentinel data has been incorporated in the model as the average mosquito count of the four houses

**Figure 2:**
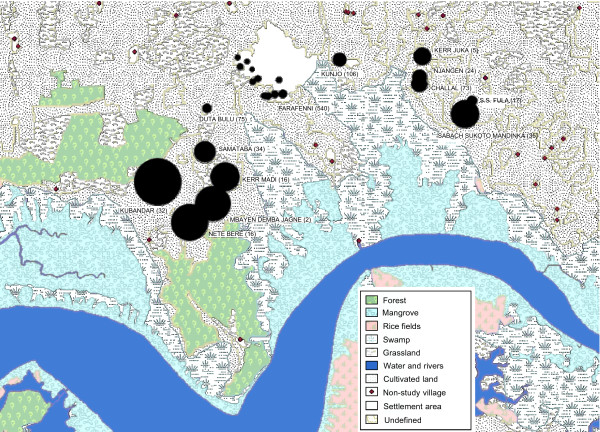
Relative abundance of *An. gambiae s.l*. in study blocks (no. of houses sampled). Circle diameters are proportional to geometric mean no. of *An. gambiae *caught/light trap/night for each block.

Multivariate analysis on the data unadjusted or adjusted for sentinel house mosquito counts gave extremely similar results. Thus the results derived from the unadjusted values are presented. The risk of finding *An. gambiae s.l*. indoors was 89% less in town than in rural homes. The spatial abundance of mosquitoes collected in different parts of the study area is shown in Figure 2.

Horses tethered next to the house at night were associated with 23% fewer mosquitoes indoors. Homes with mud brick walls were 44% more likely to have mosquitoes as were those with open eaves (37% decrease in odds ratio). Vectors were more common where there were more people (4% increased in odds ratio per person), but were less common when churai was burnt in the house (44% decrease in odds ratio).

## Discussion

Results here identify some of the many factors that govern the risk of exposure to malaria vectors, particularly at the compound and household level. The majority of mosquito breeding sites are found on the flooded alluvial plains bordering the River Gambia [4,21]. The rural settlements are close to these habitats and therefore have more mosquitoes than those situated on the edge of Farafenni town, further away from these sites [22]. However, it can be seen that the numbers of vectors in town are disproportionately less than one would expect if trap catch was simply related to distance from breeding site.

In this part of The Gambia, the river is still brackish at the beginning of the rains, but becomes less so during the season as the salt front is pushed down river by the rising water level. This explains why *An. melas*, the more saltwater tolerant member of the species complex, is often more common at the beginning of the rains than during the middle [8,23,24]. However, both *An. gambiae s.s*. and *An. arabiensis *were common in the main part of the rainy season and are the two most important vector species in The Gambia [1,25,26].

At the compound level, it was found that the presence of a horse tethered near the house at night was associated with lower numbers of mosquitoes in the trapping room, although the presence of cattle was not. This finding is of significance to mosquito control since zooprophylaxis, the use of large domesticated animals to reduce biting frequencies has been successful in some parts of the world [27]. The findings here concur with previous findings where it was shown that the presence of cattle was not associated with protection against mosquito bites [19]. On the other hand the presence of a horse was protective. In an earlier study, it was found that 32% of all indoor resting members of the *An. gambiae *complex had fed on equine blood (horses and donkeys), including *An. gambiae s.s*. (34%), *An. arabiensis *(26%) and *An. melas *(28%) [19]. Similar affinity for mosquitoes to feed on equines has also been reported from Senegal [28-30], illustrating the unusual zoophagy of *An. gambiae s.s*. in the west African Sahel. The reasons for the attractiveness of horses may be partly due to the high bed net usage in The Gambia [31] making it difficult for a blood-questing mosquito to locate a human host, as well as the unusually high density of equines in the Senegambia region [32].

Mosquitoes were more likely to enter houses constructed with mud brick walls than those with concrete walls. The most likely explanation for this is that there are more potential access points for mosquitoes in mud brick walls that crack and subside over time compared to concrete walls. These walls may also provide microenvironments conducive for mosquito survival. Mud walls have been shown to be positively associated with malaria infection in Eritrea [33], specifically increasing individual risk for parasitaemia compared with individuals living in houses of other construction materials. Similarly in Côte d'Ivoire, regular sleepers in poorly constructed temporary farm huts had an increased risk of *Plasmodium falciparum *infection [34].

Mud brick walls are also associated with thatched roofed housing that is often less well built, typically needing reconstruction every three to four years or more often if affected by flooding in the rainy season. Houses built with more expensive concrete blocks tend to be better constructed and are more commonly associated with corrugated tin roofs. It has not been established here to what extent household wealth and wall type are confounded; elsewhere the existence of mud wall types was negatively associated with use of control measures and so it seems plausible that wall type is a proxy for socio-economic status [9]. Thus there is a need for future studies to control for the potential confounding effect of household wealth.

In concrete wall houses in the study area the eaves are frequently closed in an effort to reduce the risk of the roof being ripped off by sudden gusts that lift the overhanging part of the roof. Open eaves are the main route by which *An. gambiae s.l *enters homes [35] and it is well known that closing eaves will reduce the number of mosquitoes entering a house [36,37]. Thus metal roofing would seem to be protective against mosquito house entry by association with closed eaves, as it is in lowland Kenya [38]. In north-west Burkina Faso, occupants of iron-roofed houses had a decreased risk of infection compared to residents of thatched-roof and mud-roof houses [39], yet the eaves were a less likely explanation for this because in that region almost all the houses are built with closed eaves so that the roof is directly in contact with the wall (Yé, personal communication). In contrast, metal roofing in the western Kenyan highlands was found to be associated with increased malaria risk, probably because in that region metal-roofed houses frequently also had open eaves [40].

The finding that more mosquitoes are found in rooms with higher numbers of people is unsurprising since it has been shown that odours emanating from individuals attract mosquitoes towards a human host [41]. More occupants likely increases the attractiveness to Anophelines and this factor has been associated with higher Anopheline densities [42]. The relationship between an increased number of household residents and increased malaria risk has also been demonstrated in Kenya [40].

*Churai *is a collective term for various types of incense burned over small charcoal braziers in the home. Most churai burned in The Gambia is *santango*, the bark and resin of the local tree, *Daniella oliveri*. Churai is used largely for making the home smell pleasant, but it also has repellent properties [43]. However, in a previous study it did not protect children from clinical episodes of malaria [44].

## Conclusion

Because there may be unmeasured biological or social confounders operating at a scale not explored here, caution is needed before interpreting these results as indicating causal associations. Nevertheless, in view of the fact that approximately 80% of malaria transmission occurs in the home [17], it is likely that the household factors statistically associated with risk of mosquito house-entry are important. These results may serve to direct decision making and targeted use of indoor residual spraying (IRS) as a control measure. For *An. gambiae s.l*. in this setting, particular attention must be paid to the under surfaces of roof eaves if the aim is to deter house entry. Insecticide must also be rigorously applied to the interior surfaces of mudbrick walls, where the vector may be resting, if the intention is to reduce survival of vectors entering houses. However, this study has also illustrated that risk factors operate at a range of scales, and so both household level and community-level mosquito control may well be necessary to reduce transmission in rural Africa. Such risk factors and potential confounders are important to consider when assessing the protective efficacy of interventions directed at the household level, such as house screening and IRS.

## Authors' contributions

MK coordinated the fieldwork, data collection and sample identification, and drafted the manuscript. CG carried out the PCR, PM and CS performed the main statistical analyses and helped to draft the manuscript. MJ, DC and PM participated in the design and interpretation of the study and MJ provided access to the demographic database. SWL conceived of the study, participated in its design and coordination and helped to draft the manuscript. All authors read and approved the final manuscript.
